# 

**Published:** 1916-01-22

**Authors:** 


### CONTENTS.

PAGE

**Honours and the Nursing Profession** ... ... 357

**Clinical Tests of New Drugs** ... ... ... 358

**Hospital and Institutional News** ... ... ... 359

The Board Room Atmosphere—The Saturday Fund's Figures—A Patient's Gratitude after Fifteen Years—The Disregistered Man and the War—An Endowed Ward at Cardiff—Poor-Law Relief—Sympathy and Congratulations in War-Time—Robbing the Hospitals— A Matter for Regret—The Dilemma at Bradford—Employment Forbidden to Partial Convalescents —No More German Measles—An Inquest on a Death from Measles—Unpatriotic Councillors at York—Compulsory Powers to be Applied to Consumptives—A Light on the Causation of Pellagra—The Endowment of Aberdeen Royal Infirmary—An Endowment for Research—An Important Distinction—The Latest Addition to Hospital Histories—A Ministry of Science—The Housing of Munition Workers—The Medical Examination of Recruits—This Week's Drug Market.

**Poisons and Poisoning** ... ... ... ... 365

Some Legal Difficulties Explained.

**Credulity and Credibility** ... ... ... ... 367

By a Medical Officer from France.

PAGE

**A Great Extension Scheme at Cardiff** ... ... 368

Colonel Bruce Vaughan's Enterprise and Success.

**Hospitals and the Poets** ... ... ... ... 369

II.—The Medical Career of Oliver Goldsmith.

**Parliament and the Profession** ... ... ... 370

**Passing Events and Topics** ... ... ... ... 370

**War-Time Charities** ... ... ... ... ... 371

Appeal and Accountancy Methods Examined.

**The National Relief Fund in Scotland** ... ... 372

**Textile Supplies for Hospitals** ... ... ... 373

III.—The Raw Materials : Cotton and Linen.

**Patriotism and the Nurse-Training Schools**... 375

VI.—Guy's Hospital.

**The Editor's Letter-Box** ... ... ... ... 376

**Gifts and Bequests** ... ... ... ... ... 376

**Allen and Hanburys' Bicentenary** ... ... 376

**The Book Market** ... ... ... ... ... 376

**The Institutional Worker** ... ... (Suppl.) 1

A Charity Appeal in War-Time : Answers to December Question.

Enquiries and Answers.

The Sick and in Need.

Employment and Training.

Miscellaneous.

